# A Variational Method in Out-of-Equilibrium Physical Systems

**DOI:** 10.1038/srep03454

**Published:** 2013-12-09

**Authors:** Mario J. Pinheiro

**Affiliations:** 1 Department of Physics, Instituto Superior Técnico, Universidade de Lisboa, Av. Rovisco Pais, 1049-001 Lisboa, Portugal

## Abstract

We propose a new variational principle for out-of-equilibrium dynamic systems that are fundamentally based on the method of Lagrange multipliers applied to the total entropy of an ensemble of particles. However, we use the fundamental equation of thermodynamics 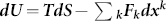 on differential forms, considering *U* and *S* as 0-forms. We obtain a set of two first order differential equations that reveal the same formal symplectic structure shared by classical mechanics, fluid mechanics and thermodynamics. From this approach, a topological torsion current emerges of the form 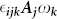, where *A_j_* and *ω_k_* denote the components of the vector potential (gravitational and/or electromagnetic) and where *ω* denotes the angular velocity of the accelerated frame. We derive a special form of the Umov-Poynting theorem for rotating gravito-electromagnetic systems. The variational method is then applied to clarify the working mechanism of particular devices.

From 1893-96, the Norwegian explorer Fridtjof Nansen, while traveling in the Arctic region, noticed ice drifting across the polar sea, at an angle of 20 to 40 degrees relative to the direction of the wind. Nansen speculated that, in addition to the force of the wind, the Coriolis Effect could be used to explain his observation. In 1905, Vagn Walfrid Ekman^1^ introduced a theory of wind currents in open seas, explaining that sea currents change direction based on their depth as a result of the Coriolis force that exists according to the rotating coordinate system associated with the Earth. In addition to atracting considerable interest in geophysical flow problems^2^, these discoveries stimulated further investigations in fields such as magnetic geodynamics, binary stars and new-born planetary systems as well as furthering work in the important problem of angular momentum transport.

The problem of enhanced angular moment transport in accretion disks^3^ and the break-down of Keplerian rotation as well as the removal of angular momentum from a vortex due to moving spiral waves, which is an important aspect of the total angular momentum balance of the core and the intensification of a tropical cyclone^4^, are all examples of problems that demand a clear understanding of the dynamics of gravito-electromagnetic rotating systems. Furthermore, special attention must be dedicated to the role of flux of angular momentum flux and its conservation. Building on the previous attempts to generate an accurate account of angular momentum transport^5^, an additional equation of conservation (besides continuity, momentum and energy equations) is required, which relates both the local angular momentum density and flux. To develop a consistent theory, the equation for the angular momentum balance must be included and, to the best of our knowledge, these problems were treated by Curtiss^6^ and Livingston and Curtiss^7^. These problems are also addressed in the present work using a variational method.

The first attempt to obtain the general equations of motion of an isolated thermodynamic system *K*, from the equilibrium condition *δS* = 0, was generated by Landau and Lifshitz^8^. Buchdahl and Simpson^9^ obtained an explicit form of the nonrelativistic motion of an isolated system in equilibrium and showed that the temperature of *K* is nonuniform when the system is accelerated. Diu et al.^10^ went a step further, but they did not attempt to build a framework to investigate the dynamics of *K* with an integrated procedure.

In this article, we develop a standard technique for treating a physical system that is based on a previously developed information-theoretic framework^11^,^12^,^13^. The proposed technique starts with the total entropy of the system composed of *N* particles (or bodies). The method of the Lagrange multipliers is then applied for the entropy differential (0-form) 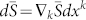 (*k* = *x*, *y*, *z*). Finally, the total entropy is inserted into the fundamental equation of entropy written using the differential form, 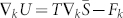, which is summed over the ensemble of *N* particles.

This method leads to a set of two first order differential equations, revealing the same formal symplectic structure shared by classical mechanics and thermodynamics^14^,^15^. When the maximization of entropy is sought, the well-known equations of (electro)dynamics (if electromagnetic entities enter into the system) result. Our method bears a resemblance to the isoentropic but non-energy-nonconserving variational principle proposed by R. Jackiw *et al.*^17^, which allows one to study non-equilibrium evolution in the context of quantum field theory but with various classical analogous, such as the Schrödinger equation, giving rise to reflectionless transmission.

This article is organized as follows. In Sec. II, we extend this mathematical formalism to non-equilibrium information theory. In Sec. III, we analyze the equilibrium and stability of a rotating plasma. In Sec. IV, we apply our formalism to angular momentum transport, obtaining, in particular, the Umov-Poynting theorem for rotating gravito-electromagnetic systems (e.g., rotating plasmas, magnetic geodynamics, vortex motion and accretion disks in astrophysics), the applications of which may contribute to the clarification of still poorly understood phenomena.

## Results

The information-theoretic method proposed in this paper constitutes an alternative approach by applying the concept of maximizing entropy to the problem of out-of-equilibrium physical systems. It bears some resemblance to the Hamiltonian formulation of dynamics, which expresses the first order constraints of the Hamiltonian, *H*, in a 2*n*-dimensional phase space, revealing the same formal symplectic structure shared by classical mechanics and thermodynamics.

Although the simplifying assumption of an isothermal system rules out its ability to accurately explain such problems as the coherent transport of angular momentum in astrophysics or certain types of laboratory devices (e.g., the Ranque-Hilsch effect), the present method attempts to further the understanding of specific trends, in particular, predicting the forced angular momentum transport that occurs radially outward from the symmetry axis of the rotation. The type of Umov-Poynting theorem obtained expresses the interplay between entropy and energy, where the energy and entropy trend towards minima and maxima, respectively, while explaining the formation of physical structures. In particular, it is that compressibility that is an important property in the transport of angular momentum and a possible driving mechanism for instability. This development is believed to be advantageous and creates options for systematic improvements.

### Mathematical procedure

Let us consider a simple dynamical system consisting of a set of *N* discrete interacting point masses *m*^(*α*)^ (*α* = 1, 2, …, *N*) with 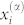 and 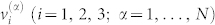 denoting the coordinates and velocities of the mass in a given inertial reference frame. The Latin subscript refers to the Cartesian components and the Greek superscript distinguishes between the different masses.

**Figure 1:**
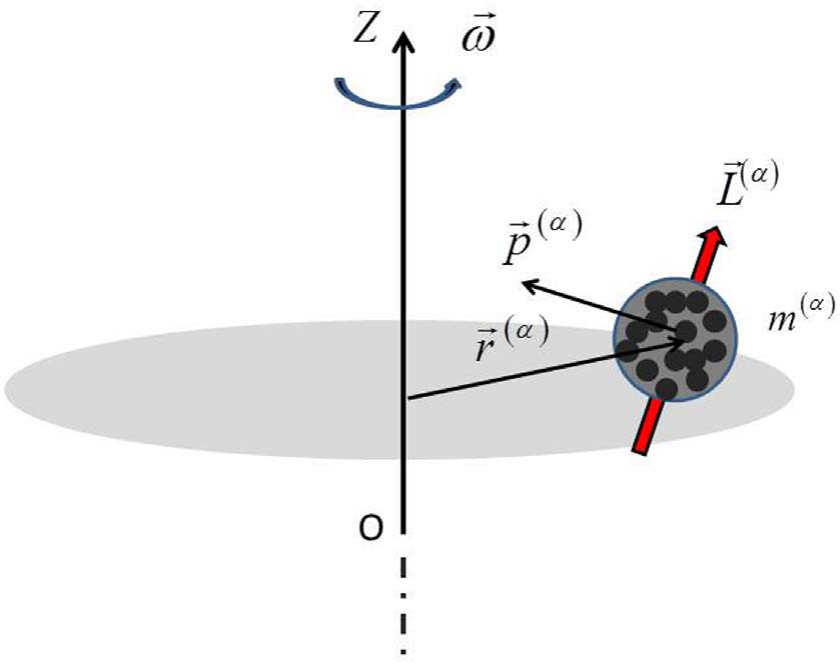
An assortment of particles of mass *m*^(*α*)^, in rotational motion around an axis OZ with angular velocity *ω*, where r^(*α*)^ denotes the position vector relative to a fixed reference frame, 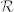, p^(*a*)^ denotes the total momentum (particle + field) and L^(*α*)^ denotes the total angular momentum of the particle.

The gravitational potential *ϕ*^(*α*)^ associated with a mass *α* is given by 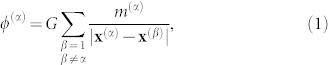
with *G* denoting the gravitational constant and **x**^(*α*)^ and **x**^(*β*)^ representing the instantaneous positions of the mass (*α*), and (*β*). Σ denotes the summation of every particle in the system. The energy, momentum and angular momentum conservation laws must be verified for a totally isolated system: 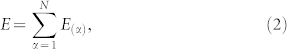

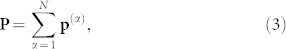
the particles' total angular momentum (the sum of the orbital angular momentum and the intrinsic angular momentum, i.e., spin) 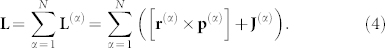
In the above equations, **r**^(*α*)^ is the position vector relative to a fixed reference frame 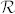, **p**^(*α*)^ is the total momentum (particle + field) and **L**^(*α*)^ is the total angular momentum of the particle, comprising a vector sum of the particle's orbital angular momentum and intrinsic angular momentum **J** (e.g., these momentums are contributed by the electron spin and/or nuclear spin, because the electromagnetic momentum is already included in the preceding term through **p**^(*α*)^), see Fig. 1. The maximum entropy principle introduces Lagrange multipliers from which ponderomotive forces are obtained.

It is necessary to find the conditional extremum; these extremum are established not for the function *S* itself, but rather for the changed function 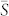. Following the mathematical procedure proposed in Ref. 13 the total entropy of the system 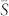 is thus given by 
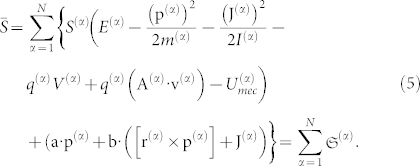
where **a** and **b** are Lagrange multipliers (as vectors). It can be shown that **v***_rel_* = **a***T* and ***ω*** = **b***T* (see also Ref. 8). The conditional extremum points form the dynamical equations of motion of a general physical system (the equality holds whenever the physical system is in thermodynamic and mechanical equilibrium), which is defined by two first order differential equations: 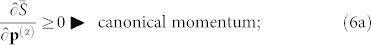

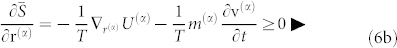


Eq. 6b gives the fundamental equation of dynamics and has the form of a general local balance equation that has an entropy gradient, ∇*_a_S* > 0, while Eq. 6a gives the canonical momentum (see also Eq. 12). At thermodynamic equilibrium, the total entropy of the body has a maximum value, constrained through the supplementary conditions 2, 3, and 4, which typically occurs as a result of the minimization techniques associated with Lagrange multipliers. In the more general case of a non-equilibrium process, according to Vanderlinde's proposition^18^, a condition required for the gravitational force to exist is that the entropic gradient in Eq. 6b must be positive. However, new physics may be developed by the set of two first order differential equations related to the interplay between the tendencies of energy and entropy to attain minima and maxima, respectively.

In non-equilibrium processes the gradient of the total entropy in momentum space multiplied by factor *T* is given by 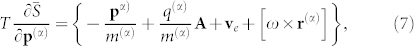
so that maximizing the entropy change in Eq. 6a leads to the well-known total (canonical) momentum: 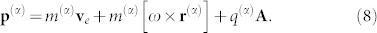
The above formulation bears some resemblance to the Hamiltonian formulation of dynamics which expresses the first order constraints of the Hamiltonian *H* in a 2*n* dimensional phase space, 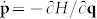 and 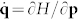. This can be solved along trajectories, such as quasistatic processes, revealing the same formal symplectic structure shared by classical mechanics and thermodynamics^14^,^15^,^16^.

The *internal mechanical energy* term, 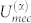, appearing in Eq. 5 may be defined by: 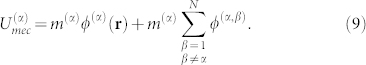
Considering this equation, by the definition of thermodynamic temperature, *∂S*^(*α*)^/*∂U*^(*α*)^ ≡ 1/*T*^(*α*)^, it follows that 
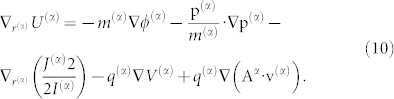
Eq. 10 contains the particle's self-energy and the particle interaction energy for the gravitational and electromagnetic fields, but it may also include other terms, such as terms included in Eq. 9, representing different occurring phenomena (exemplifying energy as a bookkeeping concept). We may recall that the entropic flux in space is a type of generalized force *X_α_*^19^,^20^; therefore, it can be shown that the following equation holds: 
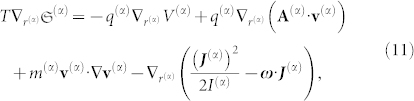
We can now write the fundamental equation of thermodynamics using the form of a space-time differential equation: 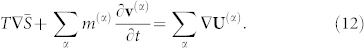
Taking into account the convective derivative, *d***v**^(*α*)^/*dt* ≡ *∂***v**^(*α*)^/*∂t* + **v**^(*α*)^·**∇v**^(*α*)^, we obtain: 
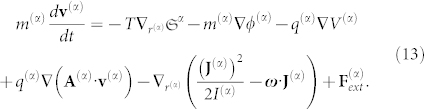
For conciseness, the term *U*^(*α*)^ now includes all forms of energy inserted into the above Eq. 5. On the right-hand side (r.h.s.), the first term must be present whenever the mechanical and thermodynamical equilibrium conditions are not fulfilled, the second term is the gravitational force, the third and fourth terms constitute the Lorentz force, the fifth term is a new term that represents the transport of angular momentum, and the last term represents other external forces that are not explicitly included but still act on the particle (*α*).

The present formalism was applied in a previous article^13^, and therein we obtained the ponderomotive forces acting on a charged particle. For a neutral particle or body in a gravitational field, Eq. 13 points to a type of extended fundamental equation of dynamics for a given species (*α*) at equilibrium and at a given point of space-time (Eulerian description): 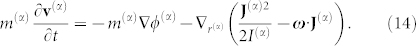
Eq. 14 gains a new term because the body possesses an intrinsic angular momentum. In a non-rotating frame of reference, we set *ω* = 0, wherein we use the work-energy theorem to obtain the total mechanical energy of the system: 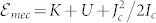. This is a common approach in classical mechanics. We are interested in the effect of a given force at a given space-time coordinate, not in its effect along the particle trajectory. It is worth noting that Eq. 14 was obtained through a variational procedure in contrast to the usual conservation theorem used, for example, in Ref. 21, 22.

Included in the internal energy term is the interpressure term (see Eq. 9; note that here we consider a homogeneous and isotropic fluid). The above described framework (see also Ref. 13 for additional information) leads us to the well-known hydrodynamic equation for a given species (*α*): 
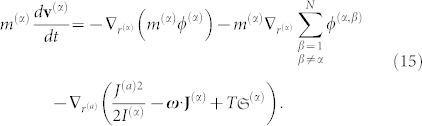
In the r.h.s. of Eq. 15, we explicitly introduce external force terms, eventually present in open systems.

Using the following correspondence from particle to fluid descriptions 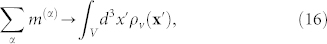
and an analogous relation for the electric charge 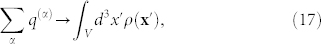
we can rewrite Eq. 15 using the form of the Euler (governing) equation: 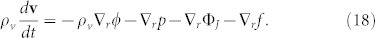
Here, as usual, the total interparticle pressure term (e.g., Ref. 23) is given by: 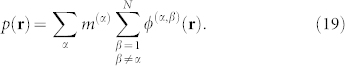
To simplify, we introduce a functional integral of the form of an intrinsic angular momentum energy density (comprising the “interaction energy term”, ***ω*** · **J**), Φ*_J_*: 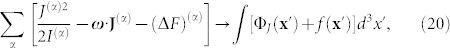
considering that the intrinsic angular momentum density refers to a given blob of fluid (with inertial momentum *I*, a measure of the local rotation, (i.e., spin, of the fluid element), and its associated free energy (per unit volume), *f* = *f*_0_ − *T_s_*. Eq. 18 also suggests that the function *S*^(*α*)^ (the *field integral of* **r**^(*α*)^) is constant along the integral curves of the space field **r**^(*α*)^. The gradient of the free energy, *f*, of the out-of-equilibrium state is the source of the spontaneous change from an unstable state to a more stable state while performing work. For example, a common source of free energy in a collisionless plasma is an electric current^24^; in a magnetically confined plasma, several classes of free energy sources are available to drive instabilities, e.g., the relaxation of a non-Maxwellian, non-isotropic velocity distribution^25^. At this stage, it is worth noting that our procedure includes the treatment of the effect of angular momentum (through Eq. 5), a necessary inclusion in a consistent theory, according to Curtiss^6^.

Furthermore, using the mathematical identity 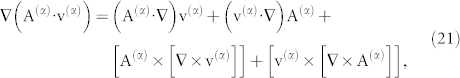
we obtain, thorugh the use of algebra, the following expression: 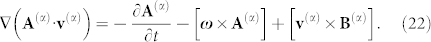
Here, **B** = [**∇** × **A**]. In addition, we notice that the following equality holds: 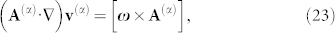
where 
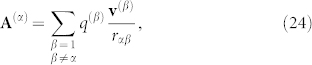
denotes the vector potential actuating on the particle (*α*) due to every other particle, and the vorticity is defined by 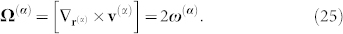
Therefore, the general equation of dynamics for a physical system (Lagrangian description) follows: 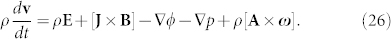
The last term in the r.h.s. of the Eq. 26 is a new term that represents a type of topological spin vector^26^, an artifact of non-equilibrium process. We will show that the topological spin vector plays a role in plasma arcs, as well as in magnetocumulative generators^27^, and suggests a new method for obtaining the helicity transport equation^28^.

## Discussion

We verify Eq. 14 using a standard example from classical mechanics: a rigid body of mass, *M*, rolling down an inclined plane of angle *θ* with the horizontal (see, e.g., p. 97 of Ref. 29). Eq. 15 can be used to solve this problem, where *ω* = 0 (the reference frame is non-rotating) and considering that only the gravitational force acts on the rolling body with an inertial moment relative to its own center of mass given by *I_c_* = *βMR*^2^. Hence, we obtain: 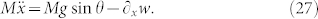
Here, 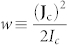. Assuming that the x-axis is directed along the inclined plane and considering that the angular momentum relative to the rigid body center of mass is given by *J_c_* = *I_c_ω*′. We find that 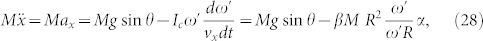
where *ω*′ = *dθ*/*dt*, while noting that *dx* = *v_x_dt* (holonomic constraint). Because *α* = *a*/*R*, we find the well-known result 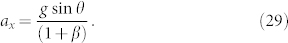
Extremum conditions imposed on the entropy or internal energy not only constraint the evolution of the system, but determine the stability of thermodynamic systems at equilibrium. Furthermore, it has been shown^30^ that a state of mechanical equilibrium can be reached, if the entropy increases with distance: 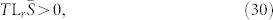
where L*_r_* is the Lie derivative along the vector field **r** acting on a scalar 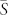. Therefore, we can find another extreme condition through the general expression 
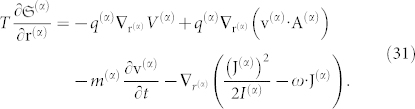
According to Noether's theorem, the total canonical momentum is conserved in a closed system. We can thus state the *closure relation*: 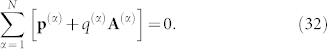
It can be shown (see Ref. 12, 13 for details) that the relation that prevails in equilibrium for a rotating plasma is given by 
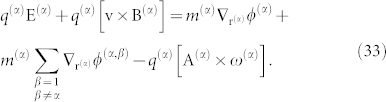
We may further develop the correspondence drawn from Eqs. 16–17 to obtain the *general condition of equilibrium of a rotating plasma* in the presence of gravitational and electromagnetic interactions (e.g., Ref. 31): 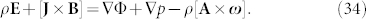
Here, to simplify the algebra, we took the averaged angular frequency ***ω***, for the entire system. We find in Eq. 34 that the vector potential **A** is presented on the same footing as the *E* and *B*-fields. The relative importance of the vector potential depends on the characteristics of the *B*-field prevalent in the system; for instance, if the *B*-field is homogeneous, the vector potential field predominates in the region near the axis because *ρωA*/*JB* ~ 1/*r*^2^. The topological spin vector term is fundamental because it produces work that is responsible for the system angular momentum modification, producing a rocket-like rotation effect on the plasma. The theoretical framework delineated here may help to clarify problems related to rotating-plasma systems^32^,^33^,^34^ and controlled thermonuclear plasma confinement^35^.

**Figure 2:**
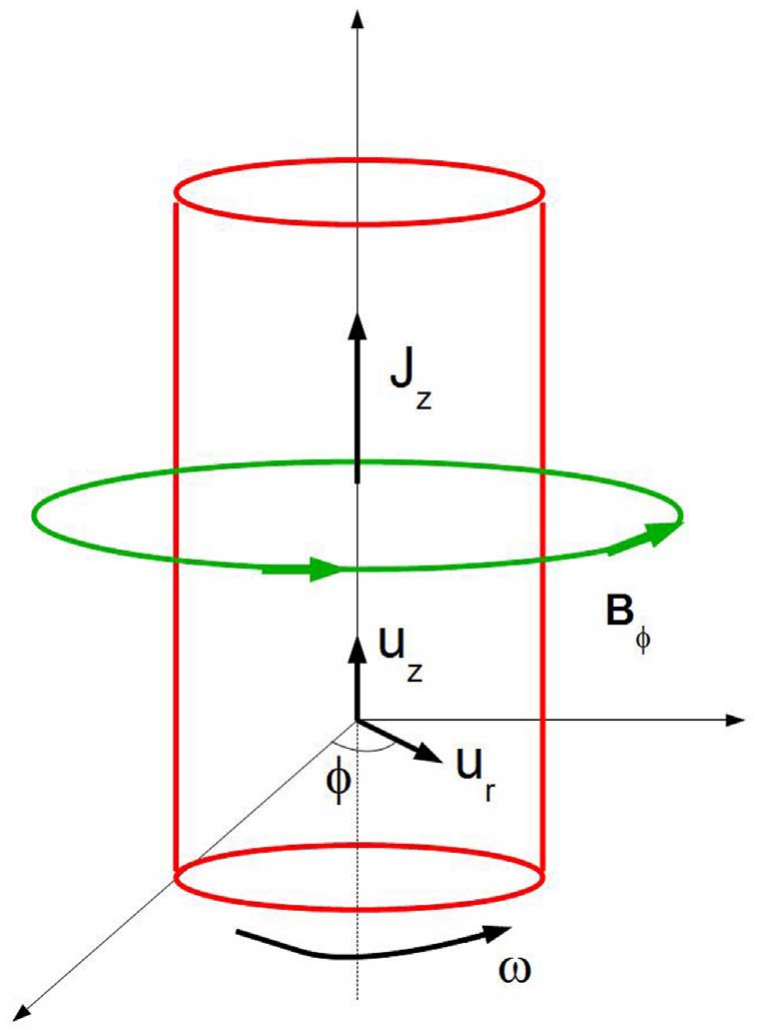
Geometry and vectorial fields in the Bennett pinch generated by an axial current *J_z_* creating a toroidal field *B_ϕ_*. If, instead, we consider a vacuum arc discharge with radial current *J_r_* and magnetic field *B_z_*, we find a rotating arc with an angular velocity *ω*.

The compression of an electric current by a magnetic field, the z-pinch effect, can be studied on the basis of Eq. 34, which gives the condition for dynamic equilibria. Let us assume a typical geometry for an infinitely long axisymmetric cylindrical arc (Fig. 2) with axial current density *J_z_* = *J_z_*(*r*). Because the current density is assumed to be constant, Maxwell's equations in the steady state yield the azimuthal component *B_θ_* = *μ*_0_*J_z_r*/2 for *r* ≤ *R* with *R* the outer boundary of the cylindrical arc. The vector potential is purely radial and is given by *A_z_*(*r*) = −*μ*_0_*R*^2^*J_z_*/4, for *r* < *R*, where the Coriolis term plays no role. We can write Eq. 34 in the form: 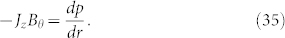
where it follows that 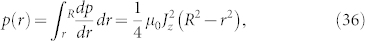
which is a well-known result.

The interaction between vacuum arcs and transverse magnetic fields is used in switching devices (see e.g. Refs. 36, 37). We can instead consider a coaxial configuration with a cathode on-axis with a stabilizing magnetic induction field **B** directed along the axis of symmetry and an arc current density **J** flowing radially (and assuming a “filamentary” current with radius *R*′, *A_r_* = −*μ*_0_*R*′^2^*J_r_*/4, with *μ*_0_ representing the permeability of the vacuum). In this case, we may apply Eq. 34 and obtain the pressure differential, from the axis to the wall (at *R*): 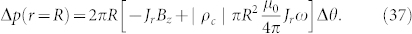

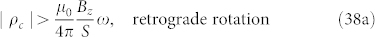

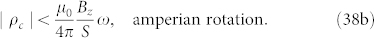
Here, *S* denotes the filamentary current cross-section, with *ρ* = *ρ_c_*. For negative charge carriers (*ρ_c_* = −|*ρ_c_*| = −*en_e_*), we obtain an Amperian (clockwise) rotation for high magnetic fields and relatively weak arc currents. We also find a retrograde rotation for higher intensity arcs (higher *S*) and small transverse magnetic fields, which is in agreement with experimental evidence (e.g., Ref. 36). Here, we see the interplay between the tendencies of the energy to attain a minimum value, while the entropy attempts to attain a maximum value. From Eq. 37, we obtain an expression for the spot velocity in a transverse magnetic field: 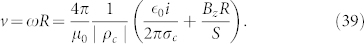
Here, we have made use of the Bernoulli relation, 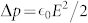, and the constitutive equation, *J_r_* = *σ_c_E*, where *σ_c_* denotes the electrical conductivity of the plasma. Although Eq. 39 is not self-consistent, it shows that the force term [**J** × **B**], is not the only important term and that the spot velocity depends linearly on the arc current (see, e.g., Ref. 44). In out-of-equilibrium systems, a new force term is present that can suppress the Amperian force under certain conditions (see also Ref. 40).

A better understanding of this phenomenon is crucial, because arc discharges are powerful generators of non-equilibrium atmospheric pressure plasmas. We compare the results predicted by Eq. 39 with experimental data available in the literature in Table 1. The calculations were done using the expression for the electrical conductivity in terms of the microscopic parameters of the plasma, 
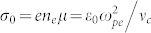
, with the electronic mobility given by *μ* = *e*/*mν_c_* (*nu_c_* denotes the electron collision frequency) because, for the majority of the data, the transverse magnetic field was below 0.1 T and is not expected to greatly influence the plasma arc electrical conductivity^41^.

We now address the transport of angular momentum, a phenomenon of great importance in several fields, such as in the working mechanism of an accretion disk, the formation of a tornado, and the planetary-atmospheric circulation.

Angular momentum conservation can be obtained using the following equation: 
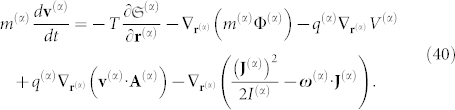
Multiplying Eq. 40 by the particle velocity 
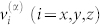
, and after rearranging the terms, we obtain: 
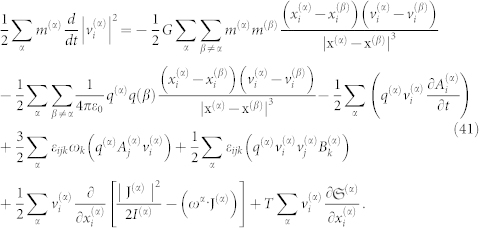
Eq. 41 can be written in a more comprehensive form if we separate the terms from their different contributions. For this purpose, we define the total kinetic energy by the expression 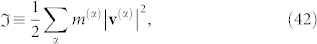
and we denote 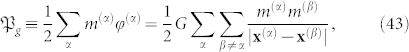
to be the overall gravitational energy. Similarly, the total electrostatic energy is given by: 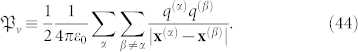
Following the Umov-Poynting theorem, it is observed that Eq. 40 reduces to the following: 
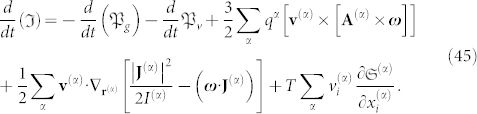
As before, it is convenient to introduce a new physical quantity that represents the rotational energy: 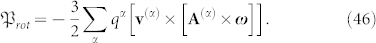
Eq. 45 can then be written using the more general form: 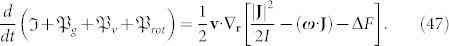
Here, **v** represents the velocity of an “element of fluid”, and the last term in brackets is an average local value. A simple analysis of Eq.47 shows that the system is rotationally stable provided the following condition is satisfied: 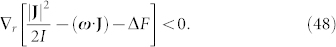
The last term is the free energy per unit volume, *F* = *F*_0_ − *TS*. It is worth noting that Eq. 48 is consistent with the Gibbs distribution in a rotating body (see, e.g., Ref. 8), which means that the radial energy flux must be positive (flowing out radially from the system's boundary). In addition, we note that the equilibrium of a gravito-electromagnetic system depends on its mechanical rotational properties as well as on the free energy available for intrinsically linking any mechanical process to thermodynamic variables and revealing options for possible unconventional mechanisms for the control of instabilities. In the domain of astrophysical plasmas, gravitational and rotational forces usually dominate the magnetic forces involved, which is a crucial aspect in the development of instabilities.

From Eq. 47, we see that equilibrium ensues (neglecting thermal and configurational effects) when the rotational velocity of the fluid satisfies the local condition (see, e.g., Ref. 45): 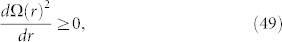
where we identify *ω* with the bulk angular velocity. Eq. 49 is related to the conservation of energy. However, when condition 48 is not fulfilled, a magneto-rotational instability (MIR)^45^,^46^ occurs, which appears as the result of the interplay between three different terms: i) the angular momentum acquired by the fluid (or particles), ii) an interaction term due to the coupling between the fluid angular momentum with the driven angular velocity, and iii) the fluid thermal energy and configurational entropy.

A typical experiment consists of a fluid rotating of a fluid between two concentric cylinders - related to the so called Taylor-Couette instability - driven by velocity gradients. In the presence of an axial magnetic field, the Taylor-Couette instability develops when Eq. 49 is not satisfied. We may also expect that, owing to the fact that for two different particle species with different inertial moment, *I^α^* ≠ *I^β^*, it can be expected that at some point, (given *r* = *r_c_* of the radial axis), an inversion of the sign of the inequality of Eq. 48 must take place, and instability occurs. In particular, in the presence of two different species with different inertial moments, the fluid may be intrinsically unstable at higher angular speeds. MIR instabilities threaten the stability of plasma configurations. In the 1960's, when MHD power plants were considered to be an efficient process for the conversion thermal energy into electrical energy, E. Velikhov, discovered this electrothermal instability, which is the cause of strong magnetohydrodynamic turbulence^45^,^47^,^48^.

Eq. 47 can be written in the form of an energy conservative equation: 
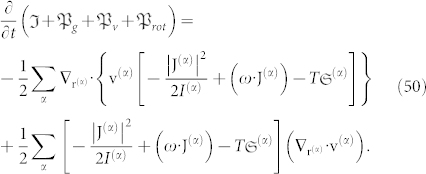
Finally, we can transform Eq. 50 into a version of *Poynting's Theorem for rotating fluids*: 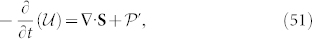
on which we define a type of *Poynting vector for rotational fluids*, which gives the rate of rotational energy flow: 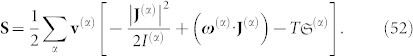
The *power input* driving the process (source/sink term) is given by: 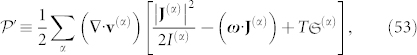
The total energy is defined by summing up the different contributions: 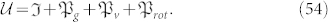
Here, the term 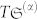 represents the thermal energy associated with the species (*α*) that is equal to −Δ*F*, the free energy of the physical system. For a system in contact with a reservoir at constant temperature this is the maximum amount of work extractable from the system; the free energy tends to decrease for a system in thermal contact with a heat reservoir. In particular, notice that when the angular velocity, *ω*, is multiplied by Eq. 40, the driving power is obtained. It is worth noting that the presence of the term ***ω*** · **J**, which plays an analogous role to the slip in electrical induction motors, that is, the lag between the rotor speed and the magnetic field's speed, is provided by the stator's rotational speed. Furthermore, we see that the power input depends on the fluid compressibility **∇** · **v**. This means that compressibility is a factor that determines the amount of transported angular momentum through the stress-tensor *τ_ij_* and may be responsible for a new driving mechanism in addition to the well-known MRI. The driving energy of the rotating system can be expressed in the form: 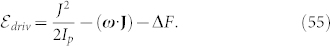
Next, we will discuss several examples illustrating the application of the variational method.

A hurricane is a natural airborne structure that converts its kinetic and potential energy by means of the transport of angular momentum from the inner core to the outer regions, conveyed either directly by moving matter, or by non-material stresses such as those exerted by electric or magnetic fields^49^. We may apply Eq. 53 to this specific problem, assuming that all of the mechanical and thermal energy is converted into electromagnetic energy *U_e_*, to obtain: 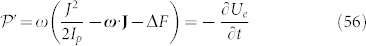
or 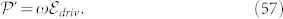
Let us consider the case of a hurricane in an axisymmetric configuration, with *J* = *ωI*. We can safely assume that 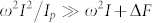. We can now envision a simple model of a hurricane with a total mass, *M*, and radius, *R*, approximated as a solid cylinder with *I* = *M R*^2^/2. Hence, the total power driving the hurricane is given by 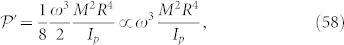
or, as a function of the fluid density *ρ*: 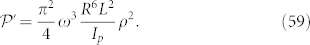
Our result shows the same type of dependency that was demonstrated by Chow & Chey^50^, and, in particular, it shows that the intrinsic inertial momentum of the particles constituting the fluid plays a substantial role.

It has been experimentally shown^51^ that periodic radiative heating of the Earth's atmosphere transmits angular momentum to it as a result of the Earth-atmosphere coupling through frictional interactions^52^. The images sent by the ESA's Venus Express confirms this fact on Venus (Earth's planetary twin) based on the presence of a “double-eye” atmospheric vortex at the planet's south pole and the presence of high velocity winds whirling westwards around the planet, which is characterized by a four-day period.

**Figure 3:**
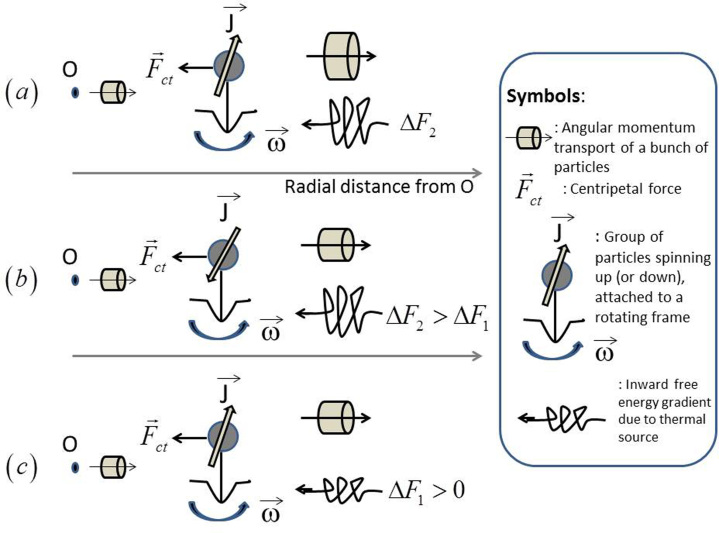
A group of particles spinning about their axes and revolving around a common axis, Oz, subject to a centripetal force. Three situations typically occur. (a) An outward transport of angular momentum occurs with a larger gradient, while free energy flows to the center of the field. (b) If the inward free energy gradient is dominant relative to the angular momentum gradient, a reversal of the particle' angular momentum may occur. (c) If the angular momentum gradient is of the same order of magnitude as in case (b) but still dominant relative to the inward free energy gradient, the particles may continue spinning in the same direction.

Schubert and Withehead's^51^ conducted an experiment with the purpose of providing an explanation for the high wind velocities during apparent cloud formation in the upper atmosphere of Venus. In this experiment, a Bunsen flame rotating below a cylindrical annulus filled with liquid mercury induced the rotation of the liquid mercury in a direction counter to that of the rotating flame. The speed of the flame was 1 mm/s and the temperature of the mercury increased from room temperature at a rate of approximately 3°C per minute. After 5 minutes, a steady-state flow was established, with the mercury rotating in the counter-direction of the flame, with a speed of approximately 4 mm/s. If we consider the liquid to be a spinning body, we can use Eq. 49 to estimate that 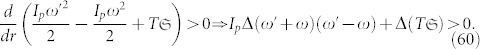
Hence, the following result is obtained: 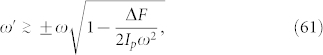
and, in the limit *ω* ≈ *ω*′, 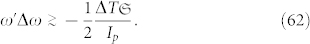
Here, *ω* is the angular speed imposed on the system (heat source), *ω*′ is the mass flow induced angular speed due to a sustained source of energy; Δ*ω* ≡ *ω*′ − *ω*. We use the following tabulated data: 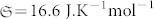 for mercury at temperature of the experiment and *ρ* ≈ 13.6 × 10^−3^ kg/m^3^. We also consider that the volume of mercury is contained in the channel of the experimental apparatus forming a rim with an average radius of *R* = 30 cm. Using Eq. 62, we estimate that after one minute, the speed must be approximately 3.7 mm/s. It is clear that the sense of rotation and the speed of the wind depend on the latent heat stored in the planetary atmosphere and the temperature difference between the boundaries (through Δ*T*). Eq. 61 is consistent with the results reported by^53^, where a maximum mean surface flow is observed corresponding to the angular velocity of the heat source when the convective velocity (in our example, 
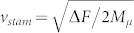
, with *M_μ_* denoting the molar mass) is attained. Note that, in fact, our *v_stam_* represents the limiting speed for the transport of angular momentum. For mercury, this velocity is *v_stam_* ≈ 24 m/s, with *M_μ_* = 200.59 g/mol in the conditions of the moving flame experiment. The existence of a limit to the amplification of the angular speed was also suggested in Ref. 54, which demonstrated the effect of a heating or cooling source in the momentum equilibration. Three possible cases for the clockwise or counter-clockwise motion of a fluid in a rotation frame are observed in Fig. 3.

Although the initial assumptions taken in the present formulation require further research and considering that the interactive terms with the medium, conveyed, for example, through the thermal diffusion coefficient, are not taken into account by Eq. 49, the agreement is reasonable and offers a possible explanation for this effect. Once again, the problem of radiative atmospheric heating reveals an interesting interplay between energy and entropy, with each attempting to achieve a different equilibrium condition.

## Additional information

PACS: 45.10.Db, 05.45.-a, 83.10.Ff, 52.30.-q, 52.80.Mg, 47.32.C-, 47.32.Ef, 95.30.Qd, 52.55.Fa

## Figures and Tables

**Table 1:** Comparing Eq. 39 with experimental data. Electric current *i* = 60 A

	Low pressure DC discharge	Atmospheric pressure DC discharge
*T_e_*(*eV*)	1.0^a^	0.87^a^
Electron density (cm^−3^)	*n_e_* = 2.5 × 10^12 ^f	*n_e_* = 2 × 10^16 ^b
Collision frequency (s^−1^)	*ν_c_* = 6.98 × 10^7^	*ν_c_* = 7.2 × 10^11 ^c
Plasma frequency (s^−1^)	*ω_pe_* = 8.94 × 10^10^	*ω_pe_* = 8 × 10^12 ^d
Average Speed (m/s)	3.0 (~ 2)^e^	10^−3^(5 × 10^−3^)^e^

^a^For laboratory discharges, the Coulomb logarithm is ln Λ ~ 10, see Ref. 38, for electron temperatures of the order of *T_e_* ≈ 10000 K^39^.

^b^Ref. 39.

^c^The frequency of collision was calculated using the standard expression 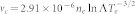, Ref. 38.

^d^We use 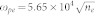, (see Ref. 38).

^e^In parenthesis are the experimental data collected for atmospheric pressure, from Ref. 40; for low pressure, see Ref. 42.

^f^Data interpolated from Ref. 43, assuming *T_e_* = 0.8 eV.
